# Chronic Pulmonary Histoplasmosis and its Clinical Significance: an Under-reported Systemic Fungal Disease

**DOI:** 10.7759/cureus.751

**Published:** 2016-08-26

**Authors:** Venkataramana Kandi, Ritu Vaish, Padmavali Palange, Mohan Rao Bhoomagiri

**Affiliations:** 1 Department of Microbiology, Prathima Institute of Medical Sciences

**Keywords:** histoplasma capsulatum, histoplasmosis, pulmonary histoplasmosis, chronic pulmonary histoplasmosis

## Abstract

Histoplasmosis is a systemic fungal mycosis caused by *Histoplasma* *capsulatum*. It is a dimorphic fungus which lives as a saprophyte in the environment and occasionally infects immunosuppressed people. *H capsulatum* is a ubiquitous fungus present throughout the globe and is more common in the temperate world. Human infection with *H capsulatum* occurs through respiratory route by inhalation of spores present in the air as droplet nuclei. Pulmonary histoplasmosis is difficult to diagnose, more so in the regions where tuberculosis is endemic, and many infected patients remain asymptomatic. In the case of immunosuppression, clinical symptoms of pulmonary infection may be seen along with chances of dissemination. We report a case of chronic pulmonary histoplasmosis in an immunocompetent individual.

## Introduction

*Histoplasma capsulatum* (*H capsulatum*) is a dimorphic fungus showing both the yeast forms (37° C) and the hyphal forms (22°  C to 30° C). It is an opportunistic fungus which usually causes infections in severely debilitated and immunocompromised individuals. Human infections with *H capsulatum* are frequently reported in cancer patients and people who underwent solid organ transplantation. In the past decade, there have been increasing reports of *H capsulatum* infection among human immunodeficiency virus (HIV), seropositive patients. Cases involving pediatric age group and elderly are also on the rising trend. *H capsulatum* is present in the environment as a saprophyte, and its association with human infection was first reported way back in 1906, by an American physician Samuel Taylor Darling. The species of *Histoplasma* causing human infections include *H capsulatum,* and *H duboisii*. *H farciminosum* is the only other species which is associated with equine infection. *H capsulatum* is present throughout the world and is considered as endemic in certain regions including the North and Latin America, Africa and some parts of Asia and Europe. The sources for human infections with *H capsulatum* are the spores present in the soil, dust arising from the demolition of old buildings, and caves. The main mode of transmission is by respiratory route (inhalation) [1-3, 4].

Human infections caused by *H capsulatum* may present as acute pulmonary histoplasmosis, chronic pulmonary histoplasmosis, cutaneous histoplasmosis, rheumatologic histoplasmosis, ocular histoplasmosis, mediastinal histoplasmosis, broncholithiasis, and progressive disseminated histoplasmosis extending to the brain. Clinical diagnosis of pulmonary histoplasmosis appears too difficult in non-endemic areas. Differential diagnosis of other microbial infections like tuberculosis, pulmonary infections caused by atypical *Mycobacteria*, legionellosis, infections caused by *Mycoplasma* spp, *Chlamydia *spp, blastomycosis, cryptococcosis, sporotrichosis, paracoccidioidomycosis, sarcoidosis, leishmaniasis, and toxoplasmosis is required [2].

It has been previously established that pulmonary histoplasmosis presents in varied forms in different individuals. Clinical manifestations depend on the immune status of the infected person and may present as acute pulmonary histoplasmosis, chronic cavitary pulmonary histoplasmosis, granulomatous mediastinitis, mediastinal fibrosis, pericarditis, pleural effusion, pneumonia, bronchiectasis, and broncholithiasis [1-3]. Dissemination to other organs including the brain, kidneys, liver, and gastrointestinal tract is also well documented among immunocompromised people. Secondary lesions involving the skin and mucous membranes have been noted as well [1-3].

We present a case of culture-positive chronic pulmonary histoplasmosis in an elderly patient with no significant underlying illness/immunosuppression to impress on the importance of fungal cultures in the laboratory diagnosis of chronic pulmonary histoplasmosis.

## Case presentation

A 60-year-old female patient presented to the medical outpatient department with a history of a cough for more than two months, intermittent fever, and dyspnea on exertion. The patient was later referred to a pulmonologist for further clinical diagnosis. Owing to the prevalence of *Mycobacterium* *tuberculosis*, a provisional diagnosis of pulmonary tuberculosis was made. A chest x-ray was done, and an endoscopy was performed. Bronchoalveolar lavage was sent to the laboratory for microbiological evaluation. Grams stain and Ziehl-Neelsen’s stain was performed. The routine bacteriological culture was done on blood agar, and MacConkey’s agar and the specimen were later inoculated into Lowenstein-Jensen’s medium for isolation of *Mycobacterium* spp. A culture for fungi was also performed on two tubes of Sabouraud’s dextrose agar (SDA), one incubated at room temperature and the other at 37° C.

**Figure 1:**
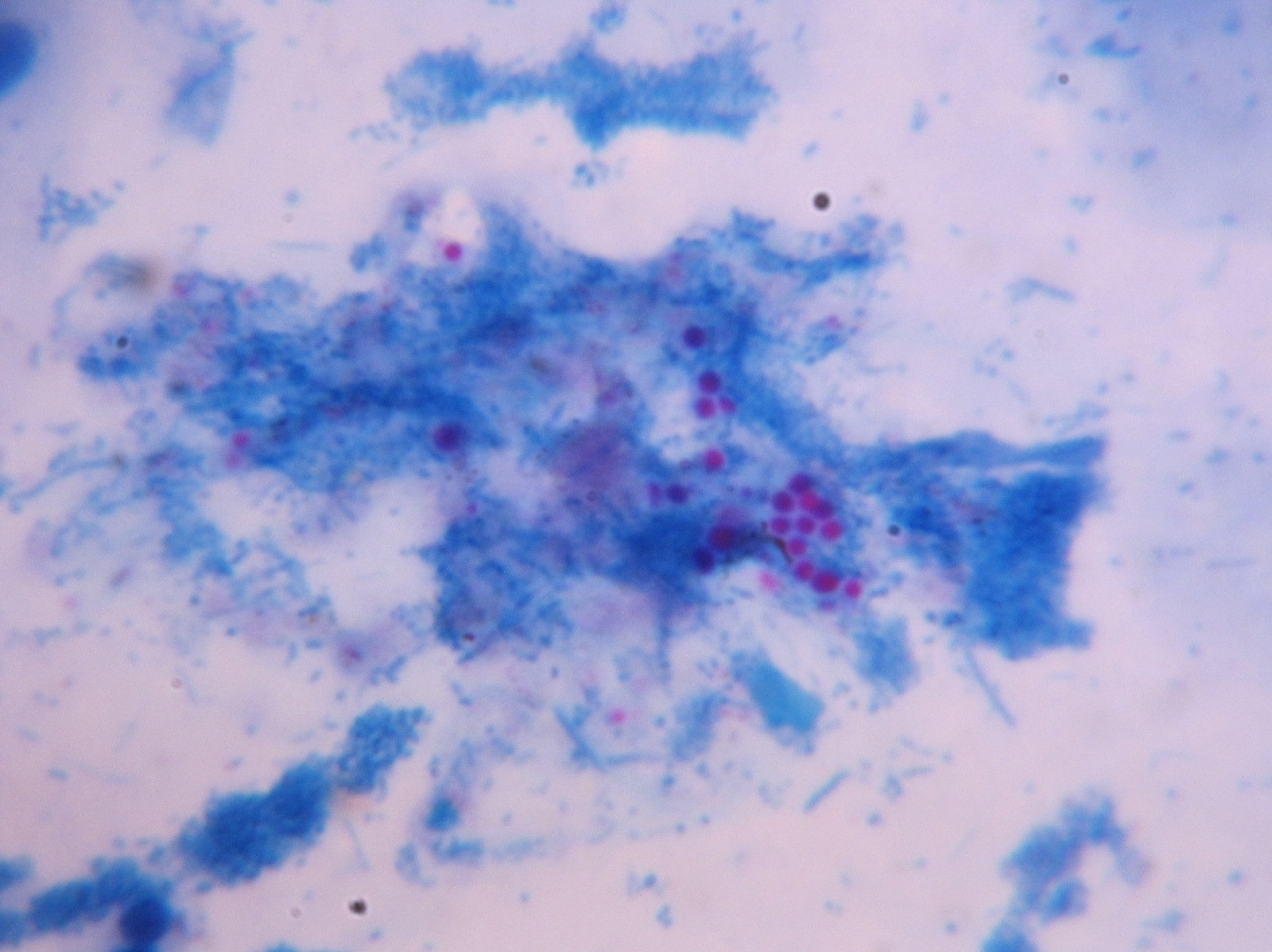
Appearance of acid-fast structures on Ziehl-Neelsen’s stain

A chest x-ray revealed multiple nodular lesions. Gram’s stain showed one to two pus cells per high power field and no bacteria. Routine culture revealed no pathogenic bacteria. Ziehl-Neelsen’s stain was negative for acid-fast bacilli but was showing the presence of spherical shaped acid-fast structures as shown in Figure 1.

**Figure 2:**
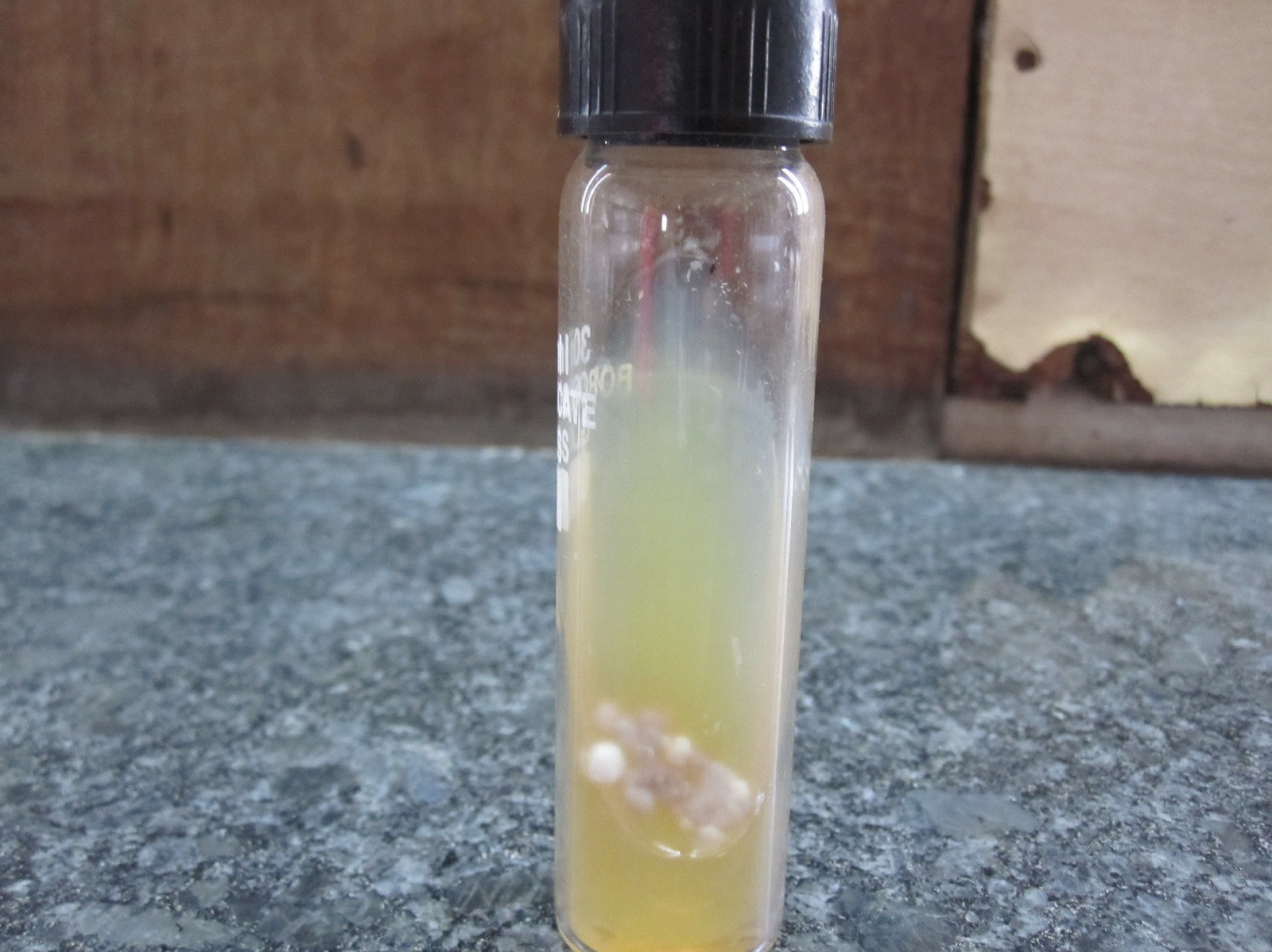
Sabourauds dextrose agar showing growth of whitish, buff colonies of Histoplasma capsulatum

Mycobacterial culture was negative after incubation for eight weeks. The fungal culture was maintained at room temperature and after two weeks revealed the growth of whitish, buff colonies which developed a central light-brownish pigmentation on further incubation as shown in Figure 2.

**Figure 3:**
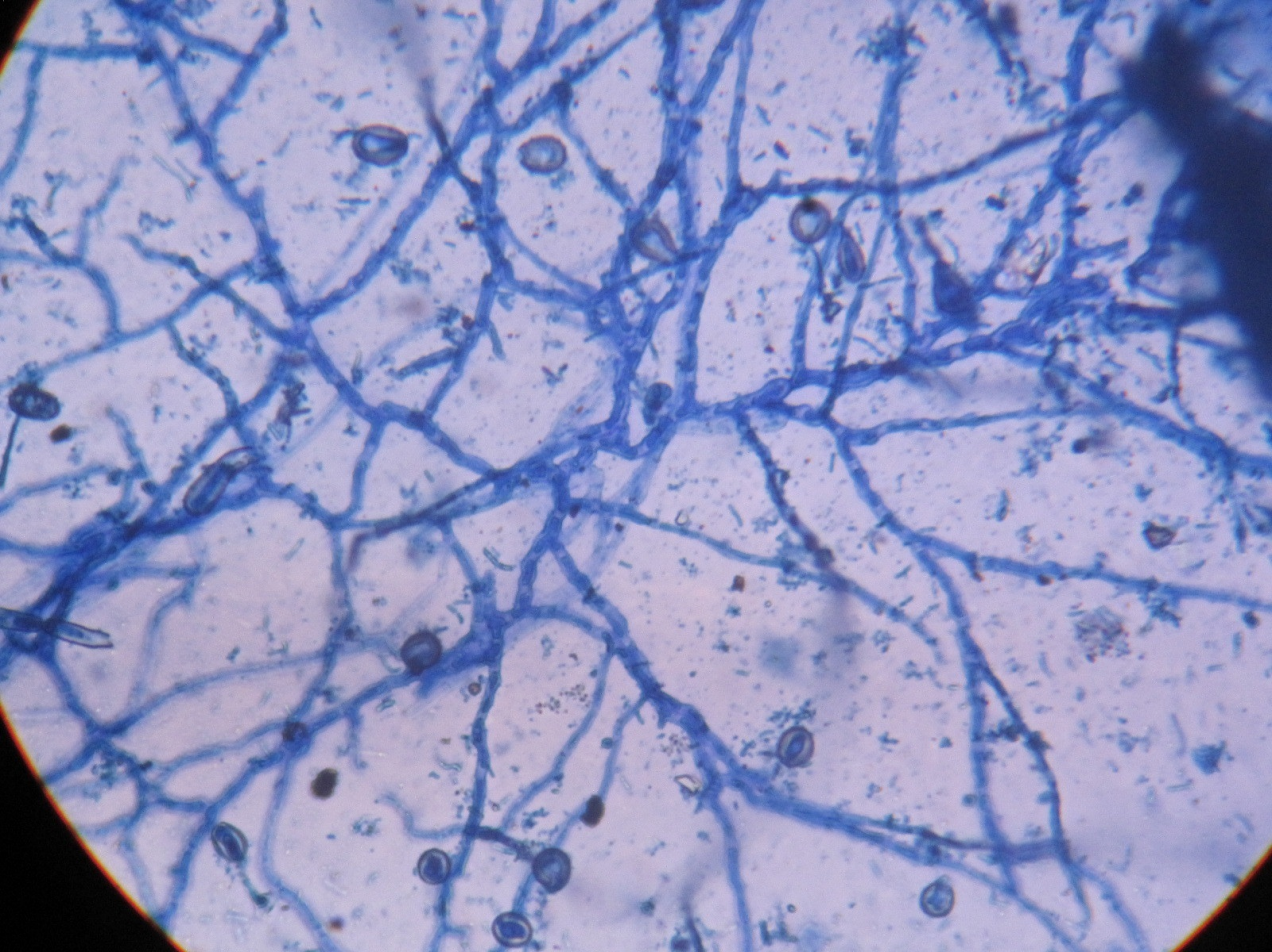
Lacto phenol cotton blue (LPCB) mount showing septate hyphae and non-tuberculate, large, thick walled macroconidia

There was no growth at 37° C. Lacto phenol cotton blue (LPCB) mount from the growth revealed the presence of hyphal forms which were septate and possessing prominent, non-tuberculate, large, thick walled macroconidia as shown in Figure 3.

The mycelia to the yeast phase (M-Y) conversion on brain heart infusion (BHI), blood agar at 370° C, showed the growth of moist, cream coloured yeast-like colonies confirming the dimorphism. Considering the fact that the symptoms lasted for more than four weeks and the patient was having no immunosuppressive condition, she was advised to take itraconazole for at least six to 12 weeks before coming back for a follow-up.

## Discussion

Histoplasmosis is a systemic fungal infection (chronic granulomatous disease) caused by dimorphic fungus *H capsulatum*. It is an intracellular pathogen affecting cells of a reticuloendothelial system including monocytes, macrophages, lymphocytes, and other defensive cells of immune system. Histoplasmosis is commonly referred to as Darling’s disease, named after its discoverer. It was first thought to be a parasitic protozoan due to its large size and intracellular presence. In the tropical regions where there is a prevalence of tuberculosis, it becomes difficult for clinicians to suspect pulmonary histoplasmosis. Mycological culture might always be required for definitive diagnosis of histoplasmosis. The infection might well remain asymptomatic in immunocompetent individuals, and progression of the disease is directly proportional to the immune status of the person. Predisposing factors for clinical infection with *H capsulatum* include patients of extreme ages (infants and elderly), recurrent respiratory tract infections, chronic alcoholism, haematological malignancies, patients who underwent solid organ transplantations, people receiving immunosuppressive drugs, patients with inborn T-cell immunodeficiency, and acquired immunodeficiency syndrome (AIDS) patients [5-6].

The present case clearly suggests the fact that pulmonary histoplasmosis may be chronic and is usually asymptomatic or subclinical, showing mild symptoms in immunocompetent people. Medical/surgical intervention and treatment is recommended only in patients with prolonged symptoms and those who are debilitated and immunocompromised. Identification of chronic pulmonary histoplasmosis and initiating treatment assumes greater significance is owing to the fact that such people might suffer from progressive pulmonary dysfunction and are at risk of developing disseminated histoplasmosis.

Early diagnosis of asymptomatic/subclinical/apparent cases of histoplasmosis assumes greater significance in non-endemic regions and immunocompromised patients. Previous studies have highlighted the importance of radiological and histopathological screening for the presence of present or previous lung infection and in identifying the cases of pulmonary histoplasmosis [7]. Recently there have been increasing reports of histoplasmosis among immunocompetent individuals [3, 8].

Laboratory confirmation of histoplasmosis greatly depends on positive mycological culture. Serological tests including the immunodiffusion, latex agglutination test, complement fixation test (CFT), and enzyme-linked immunosorbent assay (ELISA) are used for demonstration of antigens in the body fluids and antibodies in the patient’s serum. A molecular method using a gene probe is also available for confirming the diagnosis. Histoplasmin skin test for the determination of delayed-type hypersensitivity is available for epidemiological purposes and has little significance in diagnosis [9-10]. 

## Conclusions

Pulmonary histoplasmosis, although is a common disease throughout the world, it remains mostly under-diagnosed. Prevalence of other infectious agents showing similar clinical symptoms makes it difficult for physicians to suspect histoplasmosis. Serological diagnosis is not available in most laboratories present in the developing countries and is also plagued by false positivity. Molecular methods, although are available but limited only to advanced centres and remain financially constrained. In the light of all these observations, proper specimen collection, and mycological culture appear to be highly sensitive and specific for laboratory diagnosis and confirmation of chronic pulmonary histoplasmosis. Identification of asymptomatic carriage in debilitated people and initiation of effective antimicrobial therapy could contribute to a reduction in the morbidity and mortality arising from any future clinical infection.
