# Correction: Chronic Pulmonary Histoplasmosis and its Clinical Significance: an Under-reported Systemic Fungal Disease

**DOI:** 10.7759/cureus.c6

**Published:** 2016-12-12

**Authors:** Venkataramana Kandi, Ritu Vaish, Padmavali Palange, Mohan Rao Bhoomagiri

**Affiliations:** 1 Department of Microbiology, Prathima Institute of Medical Sciences

A typo appears in the first sentence of the last paragraph of the case presentation section. The original sentence reads as follows: "The mycelia to the yeast phase (M-Y) conversion on brain heart infusion (BHI), blood agar at 370° C, showed the growth of moist, cream coloured yeast-like colonies confirming the dimorphism."

"370° C" should instead read as "37° C" - thus the correct version of the sentence should read as follows:

"The mycelia to the yeast phase (M-Y) conversion on brain heart infusion (BHI), blood agar at 37° C, showed the growth of moist, cream coloured yeast-like colonies confirming the dimorphism."

